# Mutation Analysis of JAK2V617F, FLT3-ITD, NPM1, and DNMT3A in Chinese Patients with Myeloproliferative Neoplasms

**DOI:** 10.1155/2014/485645

**Published:** 2014-05-11

**Authors:** Min Wang, Na He, Tian Tian, Lu Liu, Shuang Yu, Daoxin Ma

**Affiliations:** Department of Hematology, Qilu Hospital, Shandong University, 107 West Wenhua Road, Jinan 250012, China

## Abstract

Since the discovery of JAK2V617F tyrosine kinase-activating mutation, several genes have been found mutated in myeloproliferative neoplasms (MPNs). FLT3-ITD, NPM1, and DNMT3A mutations frequently occurred in AML patients and have been found conferred with myeloproliferative neoplasms in mouse model. Therefore, we sought to search for mutations in JAK2V617F, FLT3-ITD, NPM1, and DNMT3A in 129 cases including 120 classic MPN cases and 9 MDS/MPN cases. JAK2V617F mutation was found in 60% of the 120 classic MPNs. However, none of the patients displayed FLT3-ITD and NPM1 mutations; only 2 patients harbored DNMT3A R882 mutation. Further studies including whole-genome sequence will be conducted to investigate the possible involvement of these genes in MPN.

## 1. Introduction

Myeloproliferative neoplasms (MPNs) are a class of stem cell-derived myeloid hematologic malignancies, characterized by expansion of one or more hematopoietic cell lineages with resulting bone marrow hypercellularity, a trend of transformation to myelofibrosis or acute leukemia. Mature and immature marrow elements readily traffic into the peripheral blood, as evidenced by increased white blood cells count, hemoglobin, or platelet count [1, 2]. Chronic myeloid leukemia (CML) and the three nonleukemic forms (polycythemia vera (PV), essential thrombocythemia (ET), and myelofibrosis (MF)) comprise the majority of MPNs and are commonly referred to as the classical forms [1].

The underlying causes of MPN are largely unknown. Genetic studies have identified that recurrent somatic and germline alterations may be responsible for the pathogenesis of MPN [3]. Since the discovery of the JAK2V617F mutation in 2005, an increasing number of novel somatic and germline mutations have been described in MPN in recent years, including myeloproliferative leukemia virus (MPL), TET oncogene family member 2 (TET2), additional sex combs-like 1 (ASXL1), casitas B-lineage lymphoma proto-oncogene (CBL), isocitrate dehydrogenase (IDH), and IKAROS family zinc finger 1 (IKZF1). However, none of these mutations was MPN specific, displayed mutual exclusivity, or could be traced back to a common ancestral clone [4]. Several lines of evidence suggest that mutations in genes other than these mentioned above must be present in MPN patients, and the initiating genetic events responsible for the development of MPN are still not totally understood [3].

FMS-like tyrosine kinase 3 (FLT3), a member of the class III receptor tyrosine kinase family that is expressed by early hematopoietic progenitors, plays a key role in growth regulation of hematopoietic progenitor cells [5]. Some studies have reported that FLT3 is also expressed on AML leukemic cells and stimulates survival and proliferation of leukemic blasts [6, 7]. FLT3-ITD (internal tandem duplication of FLT3) is the most prevalent mutation found in AML and has been identified in 20–30% of all AML patients [8]. Studies suggest that AML patients with FLT3-ITD have significantly elevated peripheral white blood cell counts and increased bone marrow blasts at diagnosis [9]. Moreover, Li et al. recently showed that knock-in of an ITD mutation into murine FLT3 conferred myeloproliferative disease in a mouse model [10], which indicated the potential involvement of FLT3-ITD in MPN. However, to date, the data about FLT3-ITD mutation in human MPN remain poorly defined.

The nucleophosmin 1 (NPM1, localized on 5q35) gene encodes for a multifunctional phosphoprotein located primarily in the nucleolus. NPM1 mutations are known to be common in AML and are commonly associated with a diploid karyotype [11]. Sportoletti et al. demonstrated that NPM1 acts indeed as a haploinsufficient tumor suppressor gene* in vivo* [12], while some findings suggested that NPM1 mutation in AML is likely a gain-of-function one rather than simple haploinsufficiency [13]. The most common NPM1 mutation in AML is a duplication of a TCTG tetra-nucleotide at positions 956 to 959 of the reference sequence (GenBank accession number NM_002520) and accounts for 75% to 80% of cases [14]. One “conventional” knock-in model of NPM1 mutation demonstrated that NPM1 mutation can result in myeloproliferative disease but is insufficient for leukemogenesis [13]. Nevertheless, the frequency of NPM1 mutation and its possible pathogenetic role in MPNs are rarely investigated until now.

Alterations of epigenetic markers are thought to play an important role in myeloid malignancies. In particular, aberrant DNA methylation is a hallmark of these diseases [15]. DNA methyltransferases (DNMTs) catalyze the methylation of cytosine residues of CpG dinucleotides in DNA and are encoded by the human genes DNMT1, DNMT3A, and DNMT3B [16]. More recently, a whole-genome sequencing study in AML uncovered recurrent mutations of DNMT3A in 22% of AML patients and DNMT3A mutations were associated with poor outcome [17]. Many of the reported DNMT3A mutations mainly occurred at codon R882 in exon 23, but the occurrence of DNMT3A mutations in MPN patients is not well clarified.

In the last few years, the prevalence of DNMT3A, NPM1, or FLT3-ITD has been individually studied in some myeloid neoplasms mainly including AML or MDS. Furthermore, some studies indicate that the mutagenesis of these genes may differ within different races and be associated with patient's age. Therefore, in this study, we put the three genes together, along with JAK2V617F, to determine their mutational status in a series of well-defined adult Chinese patients with MPN and explored their clinical significance; also, the association between JAK2V617F mutation and DNMT3A, NPM1, or FLT3-ITD mutation was also investigated.

## 2. Materials and Methods 

### 2.1. Patients and Samples

A total of 120 newly diagnosed classic MPN patients and 9 MDS/MPN patients according to the World Health Organization (WHO) criteria [18] were included in this study. Enrollment took place between January 2011 and December 2012 in Department of Hematology, QiLu Hospital of Shandong University, China. Bone marrow or peripheral blood samples were collected at diagnosis. The study was approved by the Institutional Review Boards of QiLu Hospital of Shandong University. Informed consent was obtained from each patient before being included in this study. The clinical characteristics of these subjects were summarized in Table 1.

### 2.2. DNA Extraction

Leukocytes were separated from bone marrow or peripheral blood samples using erythrocyte lysing solution. Genomic DNA was isolated using the TIANGEN DNA isolation kit (TIANGEN, China) according to the manufacturer's protocol. Store the DNA samples at −80°C for the following polymerase chain reaction (PCR) amplification.

### 2.3. PCR and Sequencing for DNMT3A, NPM1, and FLT3

As the recurrent mutation points, exon 23 of DNMT3A and exon 12 of NPM1 as well as FLT3 exons 11 and 12 were amplified by PCR; then the PCR products were sequenced. Briefly, PCR was performed in 25 *μ*L volume containing 12.5 *μ*L 2x Taq PCR MasterMix (TIANGEN, China), 7.5 *μ*L RNase-free ddH2O, 1 *μ*L forward and reverse primer, respectively, and 3 *μ*L genomic DNA. The primers are shown as follows: DNMT3A exon 23 forward: 5′-TCC TGC TGT GTG GTT AGA CG-3′, reverse: 5′-TAT TTC CGC CTC TGT GGT TT-3′; NPM1 exon 12 forward: 5′-GGT CTC TGT TCT TTC TGT TGA TTT CC-3′, reverse: 5′-CAA CAC ATT CTT GGC AAT AGA ACC T-3′; FLT3 exon 11 forward: 5′-GCA ATT TAG GTA TGA AAG CCA GC-3′, exon 12 reverse: 5′-CTT TCA GCA TTT TGA CGG CAA CC-3′. The amplification was done with a DNA thermal cycler (BIO-RAD S1000 Thermal Cycler). After denaturing at 94°C for 3 min, the amplification was conducted for 35 cycles at 94°C for 30 s, 55°C for 30 s, and 72°C for 1 min, followed by reextension for 5 min at 72°C.

The PCR products were loaded onto a 2% agarose gel containing ethidium bromide and electrophoretically separated. After being purified, the PCR products were directly sequenced on both directions using the ABI PRISM 3730xl analyzer (Applied Biosystems Inc., Foster City, CA, USA) to screen for the presence of mutations. The samples with mutated FLT3-ITD, mutated NPM1, or mutated DNMT3A were used as positive controls.

### 2.4. JAK2V617F Mutation Analysis

We used the TaqMan MGB probe joint real-time PCR to detect JAK2V617F mutation. The TaqMan MGB probes and Mix were purchased from Applied Biosystems. PCR amplifications of DNA were done in a total volume of 10 *μ*L PCR mixture containing 5 *μ*L TaqMan Universal PCR Master Mix, 0.4 *μ*L forward and reverse primer, respectively, 0.2 *μ*L FAM and VIC fluorophore, respectively, 2.8 *μ*L RNase-free ddH2O, and 1 *μ*L genomic DNA. The primer and TaqMan MGB probe sequences are as follows, forward primer: 5′-AAG CTT TCT CAC AAG CAT TTG GTT G-3′, reverse primer: 5′-AGA AAG GCA TTA GAA AGC CTG TAG TT-3′, probe1: FAM-TCC ACA GAA ACA TAC-MGB, probe2: VIC-TCT CCA CAG ACA TAC-MGB. PCR amplification conditions were 50°C 10 min, 95°C 30 s, 95°C 15 s, and 62°C 1 min, 45 cycles. PCR was performed with ABI 7500 Real-Time PCR system (Applied Biosystems).

### 2.5. Statistical Analysis

Statistical analysis was performed using the SPSS Statistical Analysis Software. Differences in JAK2V617F percentage, age, and peripheral blood cells counts were accessed by Chi-squared tests, Fisher's exact tests, and *t-*tests, respectively. *P* values less than 0.05 (two tailed) are considered significantly different.

## 3. Results 

### 3.1. JAK2V617F Mutation in MPN

#### 3.1.1. JAK2V617F Mutation

In the 120 classic MPNs studied, JAK2V617F mutation was found in 72/120 (60%). The frequency of JAK2V617F mutation was 76% among patients with PV (19 of 25), 60% among patients with ET (33 of 55), 37.5% among patients with MF (9 of 24), and 66.7% among MPN-u patients (10 of 15). There was significant difference between these four groups (*P* = 0.046, *P* < 0.05), and the positive incidence in PV group was remarkably higher than that in the other three groups. In addition, the only one chronic neutrophilic leukemia (CNL) patient was also observed with JAK2V617F mutation. Among the 9 MDS/MPN patients, only one had JAK2V617F mutation.

#### 3.1.2. The Association of JAK2V617F Mutation with Clinical Characteristics

As shown in Table 1, the patients with JAK2V617F mutation were much older than those without mutation (*P* = 0.000). In PV and ET group, the JAK2V617F mutant patients were older than those JAK2V617F-negative patients, while no significant difference was found in MF and MPN-u group. As shown in Table 2, compared with younger patients aged <60 years, 46.9% (30 of 64), the frequency of JAK2V617F mutation was significantly higher in older patients aged ⩾60 years, 73.1% (41 of 56) (*P* = 0.003). JAK2V617F mutation rate was higher in older patients with PV, while not in ET, MF, and MPN-u patients. In older patients, JAK2V617F mutation rate in PV patients was higher than in ET, MF, and MPN-u patients; however, this phenomenon was not seen in younger patients. As for gender, there was no statistical significance of JAK2V617F mutation incidence between male 62.5% (35 of 56) and female 57.8% (37 of 64) patients (*P* = 0.709).

We analyzed the peripheral hemogram of the 85 hospitalized patients. The number of white blood cells (WBCs), red blood cells (RBCs) and hemoglobin (HB) of JAK2V617F-positive patients was significantly higher than those of JAK2V617F-negative patients; in contrast, no significant difference was found when comparing platelet (PLT) count, while in PV patients with JAK2V617F mutation, the PLT count was higher than those without mutation. In ET and MF patients, the number of WBCs of JAK2V617F-mutated patients was much higher; besides, the JAK2V617F-mutated ET patients also had higher HB counts (as shown in Table 3).

### 3.2. DNMT3A Mutation in MPN

**Figure 1:**
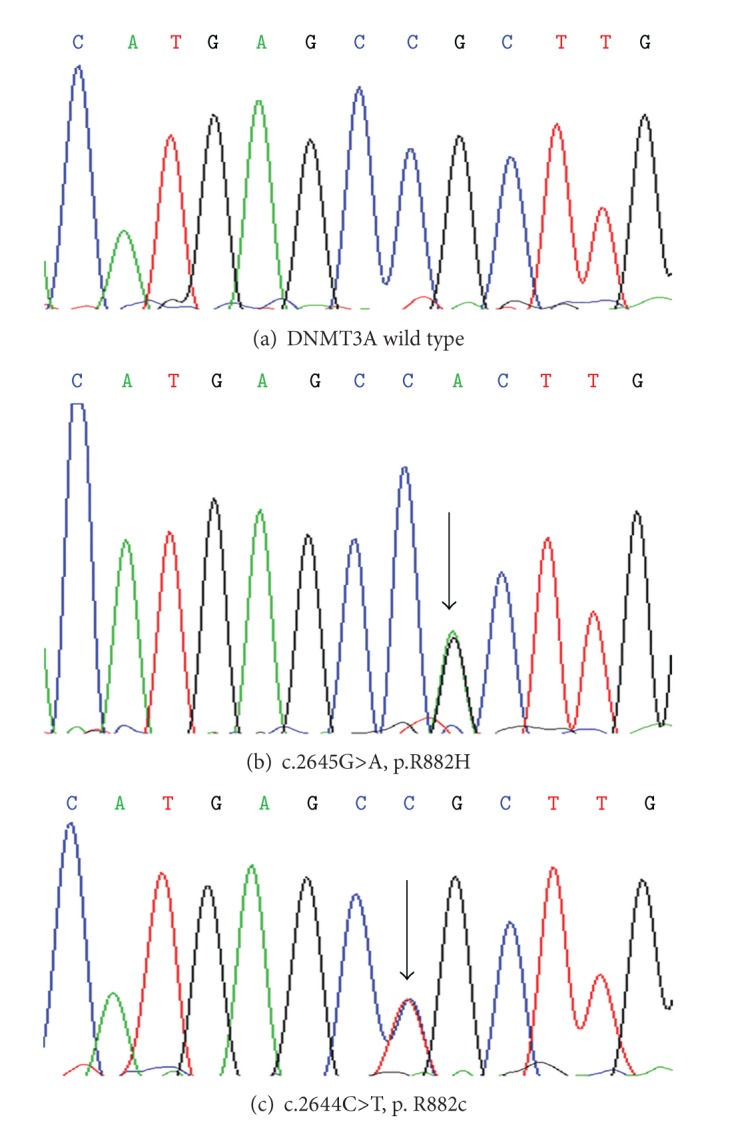
The DNMT3A mutation and wild type. (a) The DNMT3A wild type, (b) the MF patient who has a DNMT3A mutation (c.2644C>T, p.R882C), and (c) the MDS/MPN patient who has a DNMT3A mutation (c.2645G>A, p.R882H).

By sequence analysis of the DNMT3A gene, we found DNMT3A mutations in 2 patients. The two mutations were heterozygous and missense: one was a MF patient (c.2644C>T, p.R882C; JAK2V617F-positive); the other one was a MDS/MPN patient (c.2645G>A, p.R882H; JAK2V617F-negative) (Figure 1). Hence, the frequency of DNMT3A exon 23 mutation in MF was 4% (1/24), and the overall frequency in our MPN patients was close to 1% (1/120). Both the 2 DNMT3A-mutant patients were wild type for FLT3-ITD and NPM1. Moreover, the DMNT3A-mutated MF patient also had an abnormal karyotype: 46,* XY*,−3,+mar. This patient was a 60-year-old man who was diagnosed with MPN 9 years ago and later developed into postpolycythemia vera MF. And the patient also had spleen infarct complications and died ultimately with serious infections and systemic organ failure.

### 3.3. FLT3-ITD and NPM1 Mutation in MPN

**Figure 2:**
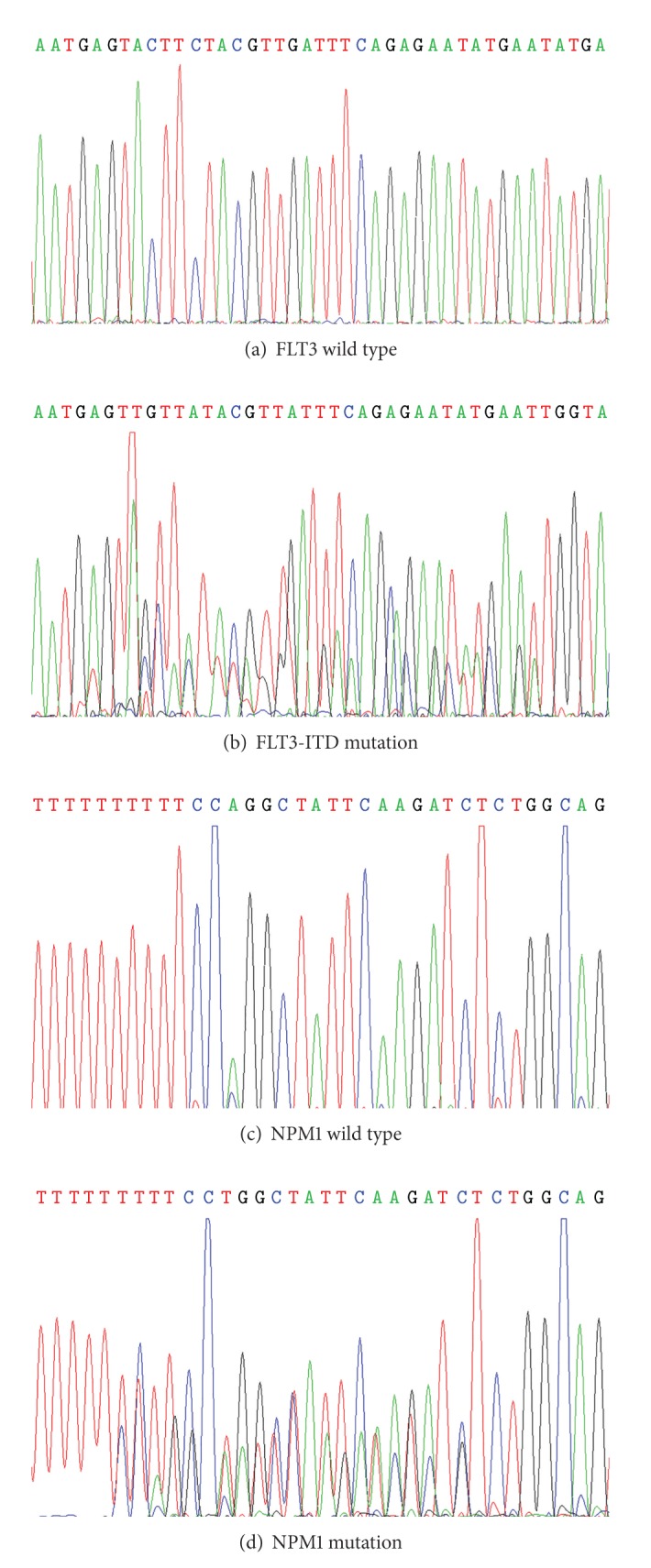
FLT3 and NPM1 wild type and mutation. The sequencing chromatograms of FLT3-ITD and NPM1. (a) FLT3 wild type, (b) the FLT3-ITD mutation in exon 11 and 12, (c) the NPM1 wild type, and (d) the NPM1 mutation in exon 12.

We analyzed the mutations of FLT3-ITD and NPM1 by PCR followed by sequencing. However, our present study did not reveal any sequence variation in the 120 MPN patients and 9 MDS/MPN patients we studied, as shown in Figures 2(a) and 2(c), while Figures 2(b) and 2(d) showed the chromatograms of positive controls (samples from AML patients with FLT3-ITD or NPM1 mutation; Figure 2(b) showed the FLT3 internal tandem duplications mutation in exons 11 and 12; Figure 2(d) showed the insertion of a TCTG tetra nucleotide at positions 956 to 959 of the reference sequence.). All these results suggested that the studied FLT3-ITD or NPM1 mutation points are unlikely the candidate factors for human MPN development.

## 4. Discussion

In this current study, we have mainly screened for the three putative candidate genes along with well-determined JAK2V617F in a cohort of Chinese MPN patients. The frequency of JAK2V617F was 60% in MPN patients. However, none of the 120 MPN patients and 9 MDS/MPN patients displayed known FLT3-ITD mutation at the juxtamembrane (JM) coding sequences and NPM1 mutation. Two kinds of mutations of DNMT3A were observed.

Amounts of molecular and clinical evidences have shown that JAK2V617F mutation has a direct causal role in the pathogenesis of MPNs. JAK2V617F mutations were found in approximately 70%–90% of patients with PV, in 35%–70% with ET, and in 30%–50% with MF, which was in line with our results. Studies have suggested that JAK2V617F mutation is more common in old than in young patients with MPN [19]. In our study, the presence of JAK2V617F was found to be significantly correlative with advanced age (≥60 years) at diagnosis. Besides, JAK2V617F mutation was common in old patients with PV. Complete blood cell count (CBC) is essential in diagnosis of MPN; several studies have showed that hemogram is altered in patients with JAK2V617F mutation. However, thus far, the relationship between JAK2V617F mutation and blood cell counts is controversial and the impact of JAK2V617F mutation on the patients' hemogram variation remains not very clear [20]. So we determined the relationship between the JAK2V617F mutation rate and hemogram in adult Chinese classic MPNs; our data demonstrated that patients harboring JAK2V617F mutation had higher leukocyte counts, red blood cell counts, and hemoglobin levels. Our JAK2V617F-mutated ET patients were found with advanced age and remarkably higher leukocytes, which was consistent with previous studies [21], while in PV and MF group, the association of JAK2V617F mutation with hemogram variations was relatively not well determined. As far as we know, our research is the first one to study the relationship between JAK2V617F mutation and hemogram as well as age factor in adult Chinese classic MPNs.

The majority of FLT3 mutations are ITDs in the JM domain encoded by exons 11 and 12 and were first reported in patients with AML in 1996 [6]. Xu et al. detected the patients with various malignant hematologic diseases and found that FLT3-ITD mutation mainly occurred in AML patients and might be a strong prognostic factor [22]. In another study, FLT3 mutations were also observed in patients with MDS or CMML, but at a much lower frequency than AML, and did not predict poor outcome [9]. However, the data about FLT3 mutations in MPN patients and their relationship with JAK2V617F mutations were limited.

Several studies in animal models uncovered the importance of FLT3-ITD in MPN; four studies have indicated that FLT3-ITD could induce myeloproliferative disease using transgenic mouse models, respectively [23–26]. Because of the putative involvement of FLT3-ITD in MPN development, we detected the total 129 MPN and MDS/MPN cases using PCR followed by sequencing method. However, no FLT3-ITD patients were found. Our negative result is similar to Pardanani et al. [27] report that no FLT3 mutations were found in a cohort of patients with chronic myeloid disorders, while being in contrast to Lin's study that FLT3 mutations occur in approximately 10% of Philadelphia (Ph) chromosome-CMPD and CMPD/MDS [5]. In a recent article, FLT3 mutation analysis was performed on 90 cases of JAK2-negative MPNs or MDS/MPNs and 62 cases of JAK2V617F-positive MPNs. One FLT3-ITD mutation was identified in the JAK2V617F-negative group (1.1%), and none were identified in the JAK2V617F-positive group, confirming the absence of FLT3 mutations in JAK2V617F-positive specimens [28], which is basically the same as our results that FLT3 mutation was rare in the usual types of MPN and the two mutations are mutually exclusive. These differences may be due to the diversity of the studied diseases or population.

NPM1 mutations, first identified by the aberrant cytoplasmic localization of NPM1 protein, were found to be frequent events in AML [14]. Some animal models bearing enforced human NPM1-mutant expression showed an expansion of hematopoietic cells and developed myeloproliferation, indicating a pathogenic role of mutant NPM1 protein in myeloid disorders [29, 30]. Some studies showed that NPM1 mutation occurred with low frequencies in patients with MDS [11, 31], while others found no mutations in MDS [32, 33]. Ernst et al. showed that NPM1 mutations occurred in 6/187 (3%) MDS/MPN patients and the 6 patients were all CMML patients, indicating that NPM1 mutation may be associated with a poor prognosis [34]. Schnittger et al. found that NPM1 mutation was observed in 6/67 secondary AML (s-AML) patients with a history of MPN and concluded that the NPM1 mutations are not only a key factor in the initiation of* de novo* AML but may contribute to s-AML following MPN [35]. However, relatively few data regarding the presence of NPM1 mutations are available for classic MPN cases. One study in a small cohort of classic MPNs (14 PV, 7 ET, and 9 MF) reported that NPM1 mutations were not observed in these patients [32]. In our relative larger cohort of Chinese MPN patients, no NPM1 mutation was found, indicating that NPM1 mutation might not be prevalent in MPN.

Mutations in DNMT3A were shown to be one of the early initiating events in AML pathogenesis [36]. DNMT3A mutations were also noted in patients with MDS and s-AML. Walter et al. found DNMT3A mutations in 8% of MDS patients, similar to the trend noted in AML. The major mutant point was at amino acid R882 and has a significantly poorer outcome [37]. The studies about DNMT3A mutation in MPN were limited and inconsistent. Stegelmann et al. reported DNMT3A mutations in 7% PV, 15% MF, and 14.3% s-AML and indicated DNMT3A alterations occurred concurrently with JAK2 [38]. Abdel-Wahab et al. delineated that total 3 DNMT3A-positive cases in 46 primary MF patients were also found to have cooccurring JAK2V617F mutation [39]. However, other studies identified that DNMT3A mutations were rare or absent [15, 40, 41]. We explored the mutation frequency of DNMT3A in 120 Chinese MPN patients, and mutation was only observed in a MF patient concurrently with JAK2V617F mutation. Moreover, this positive patient had an abnormal karyotype and spleen infarct complications and died ultimately with serious infections and systemic organ failure. Therefore, DNMT3A mutation may not be a frequent characteristic of MPN, but could be a poor prognostic indicator, always concurrently with JAK2V617F mutation. However, larger cohort of patients are needed to determine the exact frequency of DNMT3A mutations in Chinese MPN patients and to clarify its role in the molecular pathogenesis of MPN.

So far, more and more genetic events which may contribute to the pathogenesis of MPN have been elucidated. However, despite significant insight into the role of specific mutations, including the JAK2V617F mutation, the precise mechanisms in MPN remain elusive. It is very likely that additional mutations in MPN will be described soon, but practical relevance in terms of either disease prognostication or value as drug targets has so far been limited [42].

MPN genes belong to two major pathways: intracellular metabolism and epigenetic regulation [43]. The molecular pathogeneses of these three candidate genes mutations we studied here are different. (1) DNMT3A alterations are involved in epigenetic regulation of gene transcription—aberrant DNA methylation. (2) FLT3 mutation is associated with signaling pathways and proliferation. FLT3 as well as JAK2V617F abnormity can activate tyrosine kinase and result in aberrant activation of tyrosine kinase signaling [34]. (3) NPM1 mutation in exon 12 results in loss of its nuclear localization signal; the altered protein concentrates in the cytoplasm, where it dimerizes to wild-type NPM1, blocking its activity in the nucleus [43]. So burgeoning insight into the role of these genes mutations in the pathogenesis of myeloid malignancies has prompted increased interest in development of novel targeted therapies. Methyltransferase inhibitors and JAK2 inhibitors are commonly used in clinical trials; FLT3 kinase inhibitors and NMP1 targeted therapy are reported to have made exciting progress recently [44]. However, further studies that explore their precise roles in hematopoiesis and in the pathogenesis of MPN as well as their prognostic impact and potential as a therapeutic target are needed.

## 5. Conclusion

In conclusion, we first studied the mutations of JAK2V617F, FLT3-ITD, NPM1, and DNMT3A together in Chinese adult MPN patients. However, the DNMT3A R882 amino acid residue, which is a mutation hotspot in AML, was only mutated once in our series of MPNs (in a MF patient) and the other 2 mutations were not found, suggesting that these genes may be not involved in the pathogenesis of MPN, and of course this hypothesis should be further validated in the future researches, while hotspot mutations in the DNMT3A, FLT3, and NPM1 genes are not common in MPN patients maybe due to that these selected genes harbor activating mutations in other regions which were not examined. This is a limitation in our study. So further studies including whole-genome sequence will be conducted to clarify the comprehensive mutational status as well as possible involvement of these genes in Chinese MPNs.

## Figures and Tables

**Table 1:** Basic characteristic of JAK2V617F-positive and negative MPN patients.

	All patients	JAK2V617F mutation		*P* value
		Negative	Positive	
Number (%)				
All patients	120	48 (40%)	72 (60%)	
PV patients	25	6 (24%)	19 (76%)	
ET patients	55	22 (40%)	33 (60%)	
MF patients	24	15 (62.5%)	9 (37.5%)	
MPN-u patients	15	5 (33.3%)	10 (66.7%)	
CNL patients	1	0 (0%)	1 (100%)	
Age (years), $\overset{-}{x}\pm s$				
All patient	55 ± 16	48 ± 16	60 ± 11	0.000
PV patients	53 ± 23	40 ± 15	63 ± 8	0.011
ET patients	53 ± 3	47 ± 17	57 ± 12	0.016
MF patients	58 ± 12	56 ± 14	62 ± 4	0.184
MPN-u patients	53 ± 0	43 ± 20	58 ± 12	0.183
CNL patients	68	—	68	
Gender (Male/Female)				
All patients	56/64	21/27	35/37	0.709
PV patients	15/10	5/1	10/9	0.345
ET patients	20/35	8/18	12/17	0.575
MF patients	15/9	6/5	9/4	0.675
MPN-u patients	6/9	2/3	4/6	1.000
CNL patients	0/1	—	0/1	

*P* value refers to the comparison of JAK2V617F-positive versus -negative subjects.

MPN: myeloproliferative neoplasms; PV: polycythemia vera; ET: essential thrombocythemia; MF: myelofibrosis; MPN-u: MPN-unclassifiable; CNL: chronic neutrophilic leukemia.

**Table 2:** The relationship between JAK2V617F mutation and age at diagnosis.

	≥60 years *n* = (56)		<60 years *n* = (64)		*P* value
	Positive	Negative	Positive	Negative	
PV patients	14 (100%)	0	5 (45%)	6	0.003
ET patients	17 (74%)	6	16 (50%)	16	0.098
MF patients	6 (46%)	7	3 (27%)	8	0.423
MPN-u patients	4 (80%)	1	6 (60%)	4	0.6
*P value	0.016		0.471		

All patients	41	14	30	34	0.003

*P*  value refers to the comparison of JAK2V617F mutation rate between patients >60 years old and <60 years old within all patients and every subgroup.

*P value refers to the JAK2V617F mutation rate difference between four subgroups in patients >60 years old and patients <60 years old.

MPN: myeloproliferative neoplasms; PV: polycythemia vera; ET: essential thrombocythemia; MF: myelofibrosis; MPN-u: MPN-unclassifiable; CNL: chronic neutrophilic leukemia.

**Table 3:** JAK2V617F mutation and peripheral hemogram.

JAK2V617F	Number	WBC (10^9^/L)	RBC (10^12^/L)	HB (g/L)	PLT (10^9^/L)
PV	21				
Mutation	16	11.2 ± 4.5	6.8 ± 1.1	185.6 ± 32.4	362.2 ± 222.3
Wild type	5	9 ± 5.7	6.2 ± 1.7	190.2 ± 37.4	185.7 ± 71.2
*P* value		0.383	0.543	0.813	0.015
ET	35				
Mutation	20	19.4 ± 15.5	4.4 ± 0.8	133.2 ± 24.7	1046.8 ± 608.3
Wild type	15	9.3 ± 5	3.8 ± 0.8	110.1 ± 21.9	871.8 ± 296.9
*P* value		0.012	0.052	0.013	0.272
MF	22				
Mutation	8	18.7 ± 12.8	3.4 ± 1.3	83.5 ± 21.4	188 ± 51.2
Wild type	14	8.1 ± 9.2	2.6 ± 1.0	74 ± 26.5	99.9 ± 89.5
*P* value		0.035	0.179	0.372	0.135
MPN-u	6				
Mutation	4	23.8 ± 6.0	5.7 ± 2	141 ± 43.8	549.8 ± 221.5
Wild type	2	27.4 ± 17.8	3.5 ± 1.5	95.4 ± 43.3	1125.5 ± 839.3
*P* value		0.821	0.251	0.293	0.507
CNL	1				
Mutation	1	26.77 ± 0	4.22 ± 0	138.5 ± 0	233 ± 0

Total	85				
Mutation	49	17.3 ± 12.2	5.2 ± 1.7	143 ± 45.1	639.8 ± 554.4
Wild type	36	9.8 ± 8.5	3.7 ± 1.6	106.2 ± 47.2	490.4 ± 467.6
*P* value		0.002	0.000	0.001	0.197

*P* value refers to the comparison of JAK2V617F-positive versus -negative subjects.

MPN: myeloproliferative neoplasms; PV: polycythemia vera; ET: essential thrombocythemia; MF: myelofibrosis; MPN-u: MPN-unclassifiable; CNL: chronic neutrophilic leukemia; WBC: white blood cell; RBC: red blood cell; HB: hemoglobin; PLT: platelet.
